# An Analysis of Failure of Category II DOTS Therapy

**DOI:** 10.4103/0970-0218.40886

**Published:** 2008-04

**Authors:** Subodh K Katiyar, Shailesh Bihari, S Arun, Tara Rawat

**Affiliations:** Department of TB and Respiratory Diseases, Dr. Murari Lal Hospital, GSVM Medical College, Kanpur, Uttar Pradesh, India

## Introduction

Directly observed therapy short course (DOTS) is the internationally recommended strategy to ensure cure of tuberculosis; it has become the standard for the diagnosis, treatment and monitoring of tuberculosis worldwide and has been implemented in 182 of 211 countries, covering more than 77% of world's population(1) in response to the growing threat of this disease. However, while appreciating the benefits, it provides in the management of tuberculosis and it is equally important to review the shortcomings of such an important treatment strategy against tuberculosis to make it more useful. Recently, DOTS under Revised National Tuberculosis Control Programme (RNTCP) has been implemented in Kanpur district of Uttar Pradesh state. We have tried, in the present study, to analyze various reasons for failure of category (CAT) II DOTS treatment of RNTCP reporting to our department, which reveal some important facts requiring a critical review.

## Materials and Methods

The present study included patients with pulmonary tuberculosis (previously treated within RNTCP then included under CAT II) who failed DOTS CAT II treatment at various centers mainly from eastern and north-eastern parts of Uttar Pradesh and some even from surrounding states like Madhya Pradesh, Bihar and Uttarakhand. The study period extended from June 2004 to December 2005. Informed consent was taken from all subjects. All cases were critically evaluated by detailed clinical history including history of antitubercular treatment (ATT) taken before inclusion in DOTS. History regarding their socioeconomic background, smoking, alcohol intake and drug abuse was recorded and cause for their inclusion in CAT II was evaluated. Pretreatment sputum bacillary count was noted from their records. Leading questions were asked regarding non-adherence to CAT II treatment and the causes were sought into. They underwent thorough clinical examination, radiological and bacteriological evaluation. The radiographs were classified based on the report of American Thoracic Society.(2)

## Results

In all, there were 95 cases in the study [Table 1] of which 60 (63.2%) were from rural areas and 35 (36.8%) from urban areas. The average monthly income of the earning member of the patient's family in rural areas was Rs. 1512 and in urban areas was Rs. 3452. The majority of the patients (80%) were in the economically productive age group (15-44 years). Around 60% of them were illiterate or could write their name only. About half of these cases had the history of smoking (>1 year), one fifth had alcohol intake and about 5% had drug abuse history. The average numbers of household contacts (sharing the same kitchen) were seven to eight persons.

**Table 1:** Age and sex distribution

Age	Male *N* = 65 (68%) *n (%)*	Female *N* = 30 (32%) *n (%)*
15-30 years	24 (37)	16 (53)
31-44 years	26 (40)	9 (30)
≥45 years	15 (23)	5 (17)
Total	65 (100)	30 (100)

The reason for inclusion of these 95 cases in CAT II was treatment failure in CAT I (*n* = 51, 53.7%), in CAT III (*n* = 6, 6.3%), default in previous DOTS therapy (*n* = 30, 31.5%) and relapse (*n* = 8, 8.4%) [Table 2]. Thirty-two patients (26 with CAT I failure, 3 with default and 3 with relapse) were initially wrongly categorized in CAT I [Table 3]. Since they had already taken ATT for >1 month before inclusion in CAT I, they have been categorized in CAT II directly. These cases had long previous history of ATT (45 days to 9 month, mean 3 month) before inclusion in CAT I treatment.

**Table 2:** Reasons for inclusion in CAT II

	Cases	Percentage
Treatment failure in		
CAT I	51	53.7
CAT III	6	6.3
Default in previous	30	31.5
DOTS treatment		
Relapse	8	8.4
Total	95	100.0

**Table 3:** Factors contributing to failure in CAT II

Factors	Cases (%)
H/O alcoholism	22 (23)
H/O smoking	45 (47.4)
Drug abuse	5 (5.3)
Initial sputum bacillary load	
1+	22 (23)
2+	31 (33)
3+	42 (44)
Initial wrong categorization of cases in CAT I	32(33.7)
Non-adherence to CAT II	35 (36.8)

The pretreatment sputum bacillary load was 1+ in 22 cases (23%), 2+ in 31 (33%) and 3+ in 42 (44%) cases [Table 3]. Maximum number of treatment failure (84%) was observed in cases with moderate to advanced disease [Table 4].

**Table 4:** Radiographic extent of disease

Radiographic extent	Cases	Percentage
Mild	15	16
Moderately advanced	34	36
Far advanced	46	48
Total	95	100.0

There were 35 cases (37%) who had taken CAT II treatment irregularly and finally became CAT II treatment failure cases (18 cases had interrupted treatment in the intensive phase while 17 in continuation phase and 80% of the non-adherence occurred for more than 1 month). The most important causes of interruption were the lack of relief of symptoms (37%), intolerance/toxicity (17%), inability to come for drug administration during intensive phase due to loss of earning and poor general condition (15%) and migration (14%).

## Discussion

The study included 95 cases of failed CAT II (previously treated within RNTCP), who reported to our hospital for further treatment. The majority of patients (80%) were from economically productive age group (15-44 years) and from lower socioeconomic status. There were 32 patients who were initially wrongly categorized in CAT I since they had already received ATT for >1 month from local practitioners before inclusion in CAT I; they should have been categorized in CAT II directly, which could have been one of the important reason for the ultimate CAT II failure.

About 53.7% cases (*n* = 51) belonged to already failed CAT I cases and this could be one of the important reason for CAT II failure.(3) For patients with treatment failure in CAT I, if mycobacterial culture and in vitro sensitivity testing are not routinely performed, it is not possible to diagnose whether these patients are excreting multidrug-resistant bacilli. Hence management of CAT I treatment failure patients with CAT II may fail in some settings.(4) In our country, the primary drug resistance is high, hence in such a situation, treating patients who have already failed standard treatment by CAT I with simple addition of a single drug Streptomycin may lead to treatment failure once again.(3) This situation is further worsened by initial wrong categorization in CAT I. Treatment failure not only means failure of therapy but often development of acquired drug resistance as well.

High failure rates have been noticed in patients having pretreatment high bacillary load.(5) In our study also, the failure in CAT II was more when the pretreatment sputum bacillary load was higher (77% vs. 23%). Also presence of high percent (>80%) of moderate to advanced disease, mostly having cavitory lesions (which usually bear heavy bacillary load) confirms this aspect in the study.

In the present study, 35 cases of non-adherence to treatment were analyzed and important reasons were non-relief of symptoms (37%), drug intolerance (17%) and migration (14%). Another important factor was non-willingness to come thrice weekly for the drug administration (9%) during the intensive phase and most of the cases were from low socioeconomic strata and to them earning was more important than spending time at DOTS center for treatment. This factor becomes still more important when the symptoms of the disease are alleviated by initial treatment.

The average contact of tuberculosis patients was as high up to seven to eight in our study and as nucleus of Indian household is very compact and transmission of disease is easier, presence of a resistant case in such a situation increases chances of primary drug resistance.

The above study was prompted by large number of DOTS failure coming to our department and it throws some light on some of the problems of DOTS CAT II. The important causes of CAT II failure were previous CAT I failure, initial wrong categorization in CAT I, initial heavy bacillary load and non-adherence to treatment.
